# Renal biopsy in children with IgA vasculitis

**DOI:** 10.1590/2175-8239-JBN-2021-0035

**Published:** 2021-07-28

**Authors:** Mehtap Akbalik Kara, Beltinge Demircioğlu Kiliç, Mithat Büyükçelik, Ayşe Balat

**Affiliations:** 1 Gaziantep University, Faculty of Medicine, Department of Pediatric Nephrology, Gaziantep, Şehitkamil, Turkey.; 2 Gaziantep University, Faculty of Medicine, Department of Pediatric Nephrology and Rheumatology, Gaziantep, Şehitkamil, Turkey.

**Keywords:** Purpura, Schoenlein-Henoch, Biopsy, Therapeutics, Child, Treatment Outcome, Púrpura de Schoenlein-Henoch, Biópsia, Tratamento, Crianças, Desfecho Renal

## Abstract

**Introduction:**

Henoch-Schönlein purpura nephritis (HSN) is defined as Henoch-Schönlein purpura with kidney involvement, including hematuria and/or proteinuria. The aim of this study was to evaluate the data of HSN patients who underwent renal biopsy, and compare the main clinical and laboratory parameters that may affect renal biopsy findings, treatment protocols, and short- and long-term outcome of those patients.

**Methods:**

Biopsies performed in 72 HSN patients between January 2007 to January 2017 were retrospectively evaluated. They were divided into two groups according to renal biopsy classification of the International Study of Kidney Disease in Children. Renal outcome, clinical and laboratory parameters, treatment protocols, and outcome were compared between groups. Short- and long-term follow-up of patients were evaluated.

**Results:**

Of 72 patients, 47 were male (65.3%) and 44 (61.1%) were ≤10 years of age. Neutrophil-lymphocyte ratio was found higher in patients with scrotal involvement (p=0.042). Short-term unfavorable outcome was significantly higher in patients with scrotal involvement (p=0.038). Patients with hypertension and decreased creatinine clearance were found to have more unfavorable outcomes in long-term follow-up (p=0.029, p=0.040).

**Conclusion:**

Cyclosporin-A and cyclophosphamide could be effective in steroid unresponsive HSN patients. Patients with scrotal involvement, decreased creatinine clearance, and hypertension should be closely monitored for sequelae of HSN.

## Resumo

**Introdução:**

A nefrite da púrpura de Henoch-Schönlein (NPHS) é definida como púrpura de Henoch-Schönlein com envolvimento renal, incluindo hematúria e/ou proteinúria. O objetivo deste estudo foi avaliar os dados de pacientes com NPHS que foram submetidos à biópsia renal e comparar os principais parâmetros clínicos e laboratoriais que podem afetar os achados da biópsia renal, os protocolos de tratamento e o desfecho de curto e longo prazo desses pacientes.

**Métodos:**

Foram avaliadas retrospectivamente biópsias realizadas em 72 pacientes com NPHS entre Janeiro de 2007 e Janeiro de 2017. Eles foram divididos em dois grupos de acordo com a classificação de biópsia renal do Estudo Internacional de Doenças Renais em Crianças. O desfecho renal, parâmetros clínicos e laboratoriais, protocolos de tratamento e desfechos foram comparados entre os grupos. Foi avaliado o acompanhamento de pacientes de curto e longo prazo.

**Resultados:**

De 72 pacientes, 47 eram homens (65,3%) e 44 (61,1%) tinham ≤10 anos de idade. A razão neutrófilo-linfócito foi encontrada mais alta em pacientes com envolvimento escrotal (p=0,042). O desfecho desfavorável de curto prazo foi significativamente maior em pacientes com envolvimento escrotal (p=0,038). Constatou-se que pacientes com hipertensão e diminuição da depuração de creatinina apresentaram desfechos mais desfavoráveis no acompanhamento de longo prazo (p=0,029, p=0,040).

**Conclusão:**

A ciclosporina-A e a ciclofosfamida podem ser eficazes em pacientes com NPHS não responsivos a esteroides. Pacientes com envolvimento escrotal, diminuição da depuração de creatinina e hipertensão devem ser monitorados de perto para sequelas de NPHS.

## Introduction

Henoch-Schönlein purpura (HSP) is the most common self-limiting vasculitis in childhood^1^. This systemic leukocytoclastic angiitis mainly affects the small vessels of the skin, joints, gastrointestinal tract, and kidneys. However, other organs such as brain, lungs, and scrotum could also be involved during the disease^2^. It is characterized by the deposition of immunoglobulin A (Ig A)-containing immune complexes and complement components within small vessel walls and in renal mesangium^3^. The annual incidence of HSP is reported to be approximately 14-18 per 100,000 children^4^. It is more common in males and more frequently seen during winter months. Although there is often a history of upper respiratory tract infection, the etiology is unknown^5^. Palpable purpura, arthritis/arthralgia, abdominal pain, and renal manifestations are the main characteristics of the disease^6^. Henoch-Schönlein nephritis (HSN) defined as HSP with kidney involvement, including hematuria and/or proteinuria, is the most serious complication and often determines the prognosis of the patient^7^.

The aim of this study was to evaluate the data of HSN patients who underwent renal biopsy, and compare the main clinical and laboratory parameters that may affect renal biopsy findings, treatment protocols, short- and long-term outcome of those patients.

## Materials and Methods

All children with HSN undergoing renal biopsy between January 2007 to January 2017 were retrospectively evaluated. The diagnosis of HSP was defined according to the HSP criteria accepted by the European Rheumatism Association (EULAR)^8^. The signs and symptoms of 75 patients were noted and the frequency of gastrointestinal, scrotal, and renal manifestations were documented. Hemoglobin level (Hb), number of leucocytes, lymphocytes, neutrophil/lymphocyte ratio (NLR), thrombocytes, mean-platelet volume (MPV), immunoglobulin A (Ig A) and C-reactive protein (CRP) levels were determined.

Hypertension was defined as average systolic or diastolic blood pressure (BP) greater than or equal to the 95^th^ percentile for age, sex, and height according to Task Force Report on High Blood Pressure in Children and Adolescents^9^. Estimation of creatinine clearance from height and serum creatinine level was done by using the 2009 Schwartz formula (0.413x (height/serum creatinine). Low creatinine clearance was defined as an estimated glomerular filtration rate (eGFR) of <90 mL/min per 1.73 m^2^ body surface area^10^.

Nephritis was defined as the presence of microscopic or gross hematuria with or without proteinuria. Hematuria was defined as small amount of hemoglobin positivity on dipstick testing or greater than five red blood cells per high power microscopic field in a centrifuged urine. Proteinuria was defined as the presence of small amount of protein positivity on dipstick testing or nephritic (4-40 mg/m^2^ per hour or spot urine protein/creatinine: 0.2-2 mg/mg) and nephrotic-range proteinuria (greater than 40 mg/m^2^ per hour or spot urine protein/creatinine ≥ 2 mg/mg).

HSN patients were classified into five grades according to the initial presentation. Following, modified Meadow classification was used as: Grade 1, microscopic hematuria; Grade 2, persistent mild proteinuria (<20 mg/m^2^/h) and/or hematuria; Grade 3, nephritic syndrome (hematuria, decrease in eGFR, oliguria, hypertension, edema); Grade 4, nephrotic syndrome (proteinuria >40 mg/m^2^/h, hypoalbuminemia, hyperlipidemia, and edema); Grade 5, mixed nephritic-nephrotic syndrome^2^^,^ 11. Indications for renal biopsy were persistent proteinuria and/or hematuria, nephrotic and nephritic syndrome, or hematuria with renal functional impairment.

Renal biopsies, all of which contained more than ten glomeruli, were processed for light and immunofluorescence microscopy. Renal biopsies were graded from I to VI according to the classification of the International Study of Kidney Disease in Children (ISKDC), as follows: Grade I, minimal glomerular abnormalities; Grade II, pure mesangial proliferation, focal or diffuse; Grade III, crescents/segmental lesions <50%, focal or diffuse; Grade IV, crescents/segmental lesions 50-75%, focal or diffuse; Grade V, crescents/segmental lesions >75%; Grade VI, pseudo mesangiocapillary changes^12^. Immunohistochemical studies were done by using fluoresceinated monospecific antisera to human immunoglobulin IgG, IgA, IgM, C3, C1q, and fibrinogen.

Clinical outcome was graded according to Meadow's criteria: A, normal (no hypertension, urinary abnormality, and proteinuria with normal plasma creatinine concentration); B, minor urinary abnormalities (proteinuria <20 mg/m^2^/h with or without microscopic-recurrent or macroscopic hematuria); C, active renal disease (proteinuria >20 mg/m^2^/h, hypertension or elevated plasma creatinine level); and D, renal insufficiency (GFR below 60 mL/min/1.73 m^2)^. Complete remission was considered as grade A according to Meadow's criteria for favorable outcome. The findings of grades B, C, and D were considered as no remission with unfavorable outcome^2^^,^ 13.

According to the ISKDC, grade I and grade II patients were classified as group I, and Grade III to VI were group II. Gender, age, gastrointestinal, joint and scrotal involvement, presence of hypertension, clinical presentation, short-term and long-term follow up (according to Meadow's criteria), decreased creatinine clearance, Hb level, NLR, white blood cells (WBC), MPV, CRP, and Ig A levels together with patient outcome were compared between groups. All clinical and laboratory parameters were also evaluated according to short-term and long-term outcomes of patients.

The preferred initial treatment protocol was oral steroids. If patients did not respond to steroid treatment at the end of 6 to 8 weeks, cyclophosphamide or cyclosporin-A (Cy-A) was initiated. Patients who had class IV and V renal disease with renal failure were treated firstly with pulse methylprednisolone (pMP) (30 mg/kg/day for three or six consecutive days). Oral steroid was given at 2 mg/kg/day (maximum 60 mg/day) and cyclophosphamide was given at 2 mg/kg/day for 8 to12 weeks, Cy-A was given at 3-5 mg/kg/day for 12-24 months.

### Statistical analysis

Statistical analysis was performed with SPSS (Inc. Chicago Illinois USA) for Windows version 22.0. The distribution of continuous variables was evaluated by Shaphiro Wilk test. Student-t and Mann-Whitney-u tests were used to compare parametric and non-parametric data, respectively. Chi-square test was used to evaluate relationship between two categorical variables and a p value < 0.05 was considered significant.

## Results

Biopsy was performed in 75 patients with HSN. Renal biopsies were performed in 58 patients during the first month of admission, while the remaining 17 patients underwent biopsy in the second month after the appearance of HSP findings. Since 3 of 75 patients were not attended in the outpatient clinic, they were excluded from the study. Seventy-two patients evaluated for at least six months. The main characteristics, demographic features, and laboratory results of 72 patients are shown in Table 1. NLR ratio was significantly higher in patients with scrotal involvement (p=0.042). Hypertension and decreased creatinine clearance were detected in 4% (3 patients) and 5.3% (4 patients) of patients, respectively. Median eGFR was 59.27 in these four patients. The initial clinical presentation of patients are given in Table 1. Three patients with microscopic isolated hematuria had nephrotic-range proteinuria at follow-up and therefore the biopsy was performed.

**Table 1:** Demographic, clinical, and laboratory features of the patients with Henoch-Schönlein nephritis

Age (years)	9.09±3.38
Range	3-17
*≤ 10 years*	44 (61.1)
*> 10 years*	28 (38.9)
**Gender**	
*Female*	25 (34.7)
*Male*	47 (65.3)
**Follow-up period (months)**	49.58±30.01
**Other Organ/System Involvement**	
*Skin Involvement*	72(100)
*Gastrointestinal Involvement*	71(98.6)
*Joint Manifestation*	45(62.5)
*Scrotal Involvement*	7(14.8)
**Hypertension**	3(4)
**Laboratory results**	
*Increased C-reactive protein (>0.5 mg/dL)*	41 (56.9)
*Increased Ig A (>400 mg/dL)*	1 (1.4)
*Hemoglobin (gr/dL)*	12.3±1.4
*Platelet (10^3^/µL)*	393.3±102.5
*Mean platelet volume (fL)*	8.91±1.02
*White blood cell (10^3^/µL)*	11.5±4.9
*Neutrophil (10^3^/µL)*	7.74±4.4
*Lymphocyte (10^3^/µL)*	2.87±1.23
*NLR*	3.25±2.48
*eGFR <90 ml/min per 1.73 m^2^*	4 (5.3)
*Albumin (mg/dL)*	3.47±0.58
**Initial clinical presentation (Modified Meadow classification)**	
*Grade 1: Microscobic hematuria*	3(4.2)
*Grade 2: Mild proteinuria+hematuria*	44 (61.1)
*Grade 3: Nephritic syndrome*	1 (1.4)
*Grade 4: Nephrotic syndrome*	22 (30.6)
*Grade 5: Nephritic+nephrotic syndrome*	2 (2.7)
**Renal biopsy findings (ISKDC)**	
*Grade I*	3(4.2)
*Grade II*	39(54.2)
*Grade III*	28(38.9)
*Grade IV*	1 (1.4)
*Grade V*	1 (1.4)
**Meadow's Criteria (Short-term)**	
*A*	28 (38.8)
*B*	40(55.5)
*C*	4 (5.64)
*D*	0 (0)
**Meadow's Criteria (Long-term)**	
*A*	64(88.8)
*B*	7 (9.72)
*C*	0 (0)
*D*	1 (1.38)

HSN: Henoch-Schönlein purpura nephritis;NLR: Neutrophil/lymphocyte ratio; IgA: Immunglobulin A, ISKDC: International Study of Kidney Disease in Children; eGFR, estimated glomerular filtration rate. Results are presented as mean±SD and percentages (%).

In evaluation of renal biopsy, three patients (4.2%) had grade I, 39 patients (54.2%) had grade II, 28 patients (38.9%) had grade III, one patient (1.4%) had grade IV, and one patient (1.4%) had grade V findings according to the ISKDC. According to the ISKDC classification 42 patients were classified as group I (grades 1-2) and according to kidney pathology 30 patients were classified as group 2 (grades 3-5). Age, gender, follow-up duration and the other clinical, laboratory results, clinical outcome and treatment protocols according to ISKDC and comparison are given in Table 2.

**Table 2:** Comparison of characteristic features of patient groups classified according to International Study of Kidney Diseases in Children (ISKDC)

	Group 1(Grade I-II) N=42	Group 2(Grade III-IV-V) N=30	*p*
Follow-up (months) (mean±SD)	35.76±29.42	37.53±31.30	0.721
**Gender (Female/Male)**	17/25	8/22	0,225
Age			
≤10 year	25	19	0,744
>10 year	17	11	
			
Hypertension	1/41	2/28	0,370
Joint involvement	37/7	25/6	0,698
Scrotal involvement	2/40	5/25	0,120
Gastrointestinal involvement	44/0	30/1	0,233
**Laboratory results**			
Urine protein/creatinine	5.88±5.33	7.45±5.62	0.050
Albumin (mg/dL)	3.57±0.56	3.33±0.58	0.082
White blood count (10^3^/µL)	11.34±4.54	11.917±5.44	0.62
Neutrophil/lymphocyte ratio	3.09±2.51	3.48±2.46	0.526
High level of C-reactive protein (mg/dL)	24/42	17/30	0.968
Mean platelet volume(fL)	8.91±1.072	8.93±0.98	0.934
**Clinical presentation** **(Modified Meadow classification)**			0.001
Grade 1: Microscobic Hematuria	3	0	
Grade 2: Mild proteinuria+hematuria	34	10	
Grade 3: Nephritic syndrome	1	0	
Grade 4: Nephrotic syndrome	4	18	
Grade 5: Nephritic+nephrotic syndrome	0	2	
**Meadow's Criteria (Short-term follow up)**			0.001
A	26	2	
B, C, D	16	28	
**Meadow's Criteria (Long-term follow up)**			0.060
A	40	24	
B, C, D	2	6	
**Treatment**			
ACE-I/ARBs	14	16	0,090
Oral steroids *	42	30	-
Pulse+oral steroids	5	13	0,002
Cyclophosphamide	7	8	0,303
Cyclosporine	4	12	0,002

ACE-I/ARBs: Angiotensin converting enzyme inhibitor; ARBs: Angiotensin receptor blocker;

* Oral steroids had been used in all patients.

All patients received steroids as initial treatment. Patients who had class IV and V renal disease with renal failure were treated firstly with pMP and then oral steroid with an immunosuppressive drug was continued. Five patients in group I and thirteen in group II received pMP, with significant difference between groups (p=0.02). Other therapies between groups, such as angiotensin converting enzyme inhibitor (ACE-I) or angiotensin receptor blocker (ARBs), Cy-A, and cyclophosphamide are given in Table 2. When groups were compared according to kidney biopsy stages, it was seen that Cy-A was more frequently used in group II patients (p= 0.02).

Seventy-two patients were evaluated at six months of the disease (short-term follow-up) and from 12 months until the end of the disease duration (long-term follow-up). The average follow-up was 48.28 months, ranging between 12-168 months. In the short-term follow-up, according to Meadow's criteria, 28 patients (38.8%) were in grade A, 40 patients in grade B (55.5%), and four patients (5.64%) were in grade C. In the long-term follow-up, seven of the patients (9.72%) were grade B, one patient (1.38%) was grade D, while the others (88.8%) had favorable outcomes. One patient with grade B had been operated for right ureter-vesical stenosis, and the other patient with grade D had neurogenic bladder with scarring horse-shoe kidney. In the short-term follow-up, group I had much more favorable outcome when compared to group II and it was statistically significant (p=0.001). There was no significant difference in any clinical and laboratory parameters both in short and long-term follow-up results of 72 patients (Table 3 and Table 4). However, short-term unfavorable outcome was significantly higher in patients with scrotal involvement (p=0.038), while patients with hypertension and decreased creatinine clearance had more unfavorable outcomes in the long-term follow-up (p=0.029 and p=0.040, respectively).

**Table 3:** Comparison of characteristic features, laboratory findings, biopsy findings, and treatments of patient groups according to short-term outcome

	Short-term outcome		
	Favorable outcome (A)N=28	Unfavorable outcome(B-C-D) N=44	*p*
**Gender (Female/Male)**	10/18	15/29	0.888
Age			
≤10 year	16	28	0,582
>10 year	12	16	
			
Hypertension	0	3	0,277
Joint involvement	22	37	0,553
Scrotal involvement	0	7	0,038
Gastrointestinal involvement	28	43	>0,999
**Laboratory results**			
Urine protein/creatinine	6,4±5,7	6,5±5,3	0,720
Albumin (mg/dL)	3,57±0,62	3,40±0,56	0,232
White blood count (10^3^/µL)	11,06±3,6	11,9±5,5	0,899
Neutrophil/lymphocyte ratio	2,8±1,7	3,5±2,8	0,463
High level of C-reactive protein (mg/dL)	14	27	0,342
Mean platelet volume(fL)	9,1±0,9	8,7±1,0	0,170
**Clinical presentation** **(Modified Meadow classification)**			0,117
Grade 1: Microscobic Hematuria	1	2	
Grade 2: Mild proteinuria+hematuria	22	22	
Grade 3: Nephritic syndrome	0	1	
Grade 4: Nephrotic syndrome	5	17	
Grade 5: Nephritic+nephrotic syndrome	0	2	
**Treatment**			
ACE-I/ARBs	10	20	0,414
Oral steroids	28	44	-
Pulse+oral steroids	0	18	< 0,001
Cyclophosphamide	5	10	0,620
Cyclosporine	4	12	0,196

**Table 4:** Comparison of characteristic features, laboratory findings, biopsy findings, and treatments of patient groups according to long-term outcome

	Long-term outcome		
	Favorable outcome (A)N=64	Unfavorable outcome (B-C-D)N=8	p
**Gender (Female/Male)**	24/40	1/7	0.248
Age			
≤10 year	39	5	>0.999
>10 year	25	3	
			
Hypertension	1	2	0.031
Joint involvement	53	6	0.629
Scrotal involvement	5	2	0.171
Gastrointestinal involvement	63	8	>0.999
**Laboratory results**			
Urine protein/creatinine	6.0±4.8	10.4±8.5	0.146
Albumin (mg/dL)	3.57±0.62	3.40±0.56	0.343
White blood count (10^3^/µL)	11.4±4.6	12.8±7.0	0.572
Neutrophil/lymphocyte ratio	3.2±2.5	3.6±2.4	0.548
High level of C-reactive protein (mg/dL)	36	5	>0.999
Mean platelet volume(fL)	8.9±1.0	8.4±0.9	0.181
**Clinical presentation** **(Modified Meadow classification)**			
Grade 1: Microscobic Hematuria	2	1	0.014
Grade 2: Mild proteinuria+hematuria	41	3	
Grade 3: Nephritic syndrome	1	0	
Grade 4: Nephrotic syndrome	20	2	
Grade 5: Nephritic+nephrotic syndrome	0	2	
**Treatment**			
ACE-I/ARBs	26	4	0.711
Oral steroids *	64	8	-
Pulse+oral steroids	14	4	0.101
Cyclophosphamide	12	3	0.350
Cyclosporine	13	3	0.364

## Discussion

In the present study, HSN patients had renal biopsy analyzed retrospectively for 10 years. The mean age of patients was 9.09±3.38 years. The study of Sano et al. (2002)^14^ demonstrated that patients with renal involvement were significantly older than patients without renal involvement with a median age 6.7±2.4 years. Jauhola et al. (2010)^15^ showed in their study that patients with nephritis were significantly older than those without renal manifestation with a mean age 8.2±3.8 years. In this cohort, we also found that the majority of patients who performed biopsy were over 6 years of age similarly the studies conducted by the authors mentioned above^14^^,^ 15. Moreover, we divided HSN patients in two groups according to ISKDC and compared the ages ≤10 y and >10 y. No statistically significant result was obtained between groups (p=0.744).

Most series for HSP patients show that boys are at higher risk for renal involvement than girls^16^^,^ 17. However, we had only included patients with biopsies and analyzed the gender difference between groups. No statistically significant result was obtained (p=0.225). Kurt-Sukur et al. (2010)^15^ also did not find any significant gender difference between groups according to renal biopsy stages.

There are many studies in the literature comparing the presence and the severity of other organ involvement together with kidney. Although some studies demonstrated that gastrointestinal involvement is higher in patients with renal involvement, there are also studies showing no difference^13^^,^ 18 ^,^ 19. Another study suggests that 26.1% of 107 HSP patients had kidney involvement, and kidneys were more affected in patients with scrotal involvement^3^. Wang et al. (2016)^(20 )^also found a similar relationship between scrotal and kidney involvements. We found a significant association between scrotal involvement and short-term follow-up of our patients (p=0.038) and NLR was found significantly higher in those with scrotal involvement. NLR has been widely used to define the severity of inflammation^21^. Yakut et al. (2020)^22^demonstrated that HSP patients with gastrointestinal bleeding had significantly higher NLR than those without gastrointestinal bleeding. Ozturk et al. (2016)^23^ also concluded that high NLR is related to gastrointestinal bleeding and renal involvement. Considering the above findings together with ours, the higher rate of NLR may be attributable to disease severity and inflammation. However, there is no study in literature showing the relationship between NLR and scrotal involvement in HSP. Moreover, we could not find any relationship between scrotal involvement and renal outcome. Huang et al. (2018)^24^ described 698 HSP patients followed for a median of 54 months, of whom 31 patients (4.6%) had developed end-stage renal disease (ESRD). The authors concluded that baseline urinary protein, renal insufficiency, glomerular sclerosis, and tubular atrophy/interstitial fibrosis were independent predictors of renal outcomes. Both the average mean arterial pressure and proteinuria during follow-up influenced the renal prognosis. Coppo et al. (2006)^25^ found that renal functional impairment, proteinuria, hypertension, and crescentic nephritis were significantly related to unfavorable outcomes. In our study we also found a significant correlation between hypertension and renal impairment with unfavorable outcomes. Unfortunately, we could not compare the pathological findings according to the new semi-quantitative classification with renal outcome.

Renal involvement has been reported to occur in 20-50% of children with HSP; among these, 1-7% could suffer from ESRD, and the incidence of progression to ESRD is higher in adults than in children^2^^,^ 26 ^,^ 27. Generally it has been concluded that short and long-term outcomes are good and patients recover from the disease without sequelae and the risk for ESRD is very low^28^.

Despite the high incidence of HSP, long-term outcome results are poorly explained in the literature^2^. The paper by Pillebout et al. (2002)^29^ retrospectively described a cohort of 250 adult HSP patients with a median of 14.8 years of follow-up and they have found ESRD in 11% patients, while 13% had severe (creatinine clearance <30 mL/min) and 14% had moderate renal insufficiency (creatinine clearance <50 mL/min). Similarly, Ronkainen et al. (2003)^30^ reported that all patients with HSP and severe renal symptoms at onset need long-term follow-up. Koskimies et al. (1981)^31^^)^ point out 114 unselected HSP patients followed for 3-14 years, of whom only 39 had renal disease and only one patient developed ESRD. Also there is an ongoing debate about which risk factor is mostly related to HSN and ESRD. Coppo et al. (2006)^25^ revealed that the risk of progression of HSN associated with increasing proteinuria level during follow-up was greater than the risks associated with decreased renal function, severe proteinuria, hypertension, or crescents present at onset. The different rates of ESRD in literature may be related to the differences in follow-up time. Patients were followed up for almost 4 years (median 48.2 months) in our study. Only one patient suffered from stage III chronic kidney disease (CKD) (1.38%). This patient had neurogenic bladder with scarring horse-shoe kidney and he already had nephritic range proteinuria because of scarring kidney at the time of diagnosis. Therefore, it is hard to suggest that CKD may only be attributable to HSN.

Treatment of HSN may vary depending on the experience of the centers and the severity of the disease. Corticosteroids and multiple combined agents, including immunosuppressive drugs such as Cy-A and cyclophosphamide have been used^32^. In a randomized trial, 11 patients were treated with Cy-A demonstrating its efficacy with a 100% resolution of nephrotic-range proteinuria and 100% renal survival rate^33^. Park JM et al. (2011)^34^ treated 23 of the 29 HSN patients with only one course of CyA treatment and achieved stable remission after a mean follow-up of 3.2 years. The ideal duration of Cy-A treatment for HSN patients is not known. Although it varies depending on the course of disease, the authors planned the duration of treatment of one year on average in this study. In a clinical trial by Ronkainen J et al. (2003)^35^, 7 patients were treated by Cy-A for 1.7-2.1years. In our study, 15 patients were treated by Cy-A for a mean of 21.5 months. All of them were carefully followed during the treatment in which the blood Cy-A level was maintained at 50-150 ng/mL. Because of CyA-dependency, we performed a follow-up renal biopsy in only one patient and she did not have prominent CyA-induced nephrotoxicity. After a mean period of a year from the second biopsy, she had completely improved within the next six months and Cy-A was withdrawn safely. Cy-A was well tolerated by all patients, and no cases of opportunistic infection, gingival hyperplasia, hypertension, or neurologic toxicity were observed. There was mild hirsutism only in three patients (20%). Kawasaki et al. (2004)^36^ suggested that methylprednisolone and urokinase therapy combined with cyclophosphamide was useful in patients with severe HSN. Oral prednisolone combined with eight weeks cyclophosphamide therapy caused complete remission in nine Japanese children^37^. There are also some case reports of treatment with pulse cyclophosphamide in HSN patients^38^. Fifteen of the HSN patients in this study were treated by oral cyclophosphamide for 8-12 weeks, and two patients treated by pulse cyclophosphamide. These two patients taking pulse cyclophosphamide were not included into the study because of non-regular admission for follow-up after the treatment. Although we used more frequently Cy-A in group II patients (p=0.02), complete remission was achieved within 3-6 months after beginning cyclophosphamide or Cy-A in all patients and no significant difference was found in effects of both drugs on short- and long-term results (p=0.680, p=0.999).

There is little data on renal biopsy in HSN. Although this study is important in this manner, prospective randomized controlled trials with larger samples of patients would be ideal.

## Conclusion

In conclusion, HSP has a good prognosis in childhood, but some patients may have severe renal involvement. Our results suggest none of the patients whose diagnosis of HSN was confirmed by biopsy progressed to end-stage renal disease. Clinical or laboratory parameters were not available to predict the short and long-term follow up results. However, patients with scrotal involvement, decreased creatinine clearance, and hypertension should be closely monitored for clinical outcomes. Both Cy-A and cyclophosphamide could be effective in steroid-unresponsive HSN patients. Future randomized controlled trials will provide indications of better treatment choices, and may identify clinical and histological prognostic factors in prediction of kidney outcomes.
